# Effects of continuous nursing on gastrointestinal function with ileal cystostomy

**DOI:** 10.3389/fsurg.2025.1531454

**Published:** 2025-10-21

**Authors:** Jinyuan Zhou, Xushu An, Xueting Chen, Dexin Ding

**Affiliations:** Department of Urology, Affiliated Tumor Hospital of Harbin Medical University, Heilongjiang, China

**Keywords:** continuous nursing, ileal cystostomy, gastrointestinal function, quality of life, cancer

## Abstract

**Objective:**

The importance and value of continuous care for patients after ileal cystostomy is established, and relevant experience to provide references for clinical practice is summarized in this study.

**Methods:**

This study was conducted at the Affiliated Tumor Hospital of Harbin Medical University, China, from July 2020 to July 2022. A total of 40 subjects were divided into a routine nursing group, which received routine perioperative health education, and stoma nursing was given at the bedside after the operation and followed up within one week after discharge, and a continuous nursing group, which was given continuous nursing based on routine nursing measures. The two groups’ quality of life (QoL), stoma complications, Patient satisfaction, gastrointestinal function, and other indicators were evaluated.

**Results:**

All the scores linked to the QoL in the continuous nursing group were markedly better than that in the routine nursing group, and the divergence was statistically notable. The continuous nursing group had fewer stoma complications, higher Patient satisfaction, and better gastrointestinal function.

**Conclusion:**

Continuous nursing can markedly enhance patients’ QoL, lessen complications, and enhance patients’ satisfaction and gastrointestinal function. It has significant value and practical importance.

## Graphical Abstract

**Figure:**
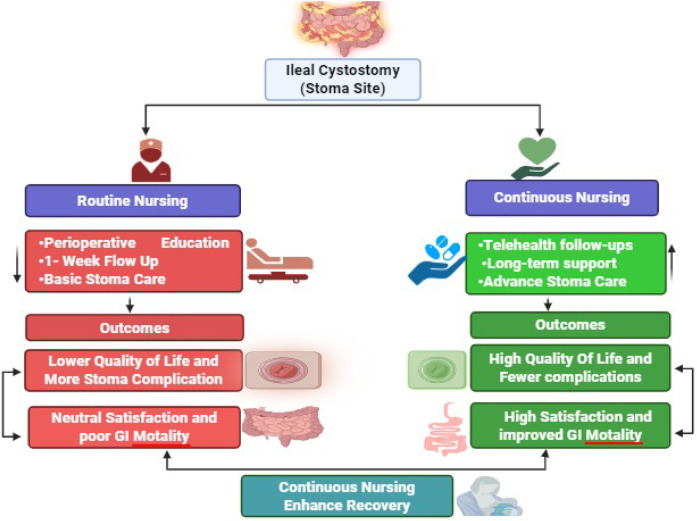

## 1 Introduction

Bladder Cancer is a common malignant tumor of the urinary system; its incidence and mortality rate have gradually increased in China in recent years due to several factors (1, 2). Similarly, gastrointestinal cancers, including colorectal, stomach, liver, and esophageal cancers, account for approximately 26% of all cancer cases worldwide and 35% of all cancer-related deaths (3). There is no unified opinion on the diagnostic criteria of bladder cancer in China. Therefore, choosing the treatment method for patients with different pathological stages and poor prognosis is significant. With the deepening of Research, various new technologies have been applied to clinical practice (4, 5). According to statistics, at present, urothelial carcinoma is the most common histological variant of bladder cancer in China, and its clinical type can also be subdivided into superficial bladder cancer and myometrial invasive bladder cancer. The most commonly used surgical method for myometrial invasive bladder cancer is radical cystectomy plus urinary diversion (6–9). After cystectomy, the abdominal wall of the ileal bladder or ureteral skin stoma is the diversion of urine flow to the abdominal wall after the operation, and it is necessary to permanently wear a pocket, which makes the Patient bear greater psychological and physiological pressure, and also brings serious negative impact on work, living, and family. Such patients need physiological and psychological nursing intervention when they are discharged from the hospital because this kind of nursing is very professional, Professional personnel are required to provide guidance and assistance (10–12). However, the current Patient health education is limited to the period of hospitalization, the community nursing strength is weak, and the responsibilities and functions can't meet the needs of patients. In recent years, the concept of continuous nursing has emerged as a promising approach to bridging the gap between hospital care and home management, particularly for patients undergoing ileal cystostomy (13). Ileal cystostomy is a type of urinary diversion surgery in which a segment of the ileum is used to create a conduit for urine to pass from the kidneys to an external stoma (14). This surgical integration of the gastrointestinal and urinary systems raises concerns regarding microbial co-residence. The ileum naturally hosts a dense population of bacteria, which are not entirely eliminated during surgical preparation. When used in urinary diversion, this segment continues to harbor intestinal microbiota that may interact with urinary tract bacteria, potentially increasing the risk of cross-contamination and postoperative infection (15). These microbial dynamics highlight the importance of continuous nursing, as structured follow-up and hygiene education may help reduce infection risks through early detection and timely intervention (16). Recent studies have demonstrated that continuous nursing interventions that are characterized by ongoing patient education, regular follow-ups, and integrated psychological support can significantly enhance patient-reported outcomes. For example (13), reported that continuous nursing strategies markedly improved self-management and reduced post-operative complications in patients requiring stoma care. These underscore the superiority of continuous nursing over conventional, routine nursing practices, which typically provide only limited, short-term educational support and follow-up care. Continuous care provides continuous care services in various forms, which can promote patients' recovery and improve their quality of life (QoL) (17–19). In this study, patients who received radical cystectomy plus ileal bladder surgery in the Department of Urology of our hospital from July 2020 to July 2022 were divided into groups for continuous nursing and follow-up the significance and value of continuous nursing for abdominal wall stoma after ileal bladder surgery were evaluated. This study aims to evaluate the effectiveness of continuous nursing in improving QoL, reducing post-surgical complications, and enhancing patient satisfaction in individuals undergoing ileal cystostomy. Additionally, it seeks to compare the outcomes of continuous nursing with routine nursing care to determine its clinical significance.

### 1.1 Data and methods

#### 1.1.1 General information

In this study, the patients who underwent ileal cystostomy in our hospital from July 2020 to July 2022 were selected as the study subjects. The inclusion criteria were: The patients diagnosed with myometrial invasive bladder cancer had no distant metastases and were able to take care of themselves before the operation. The surgical method was radical ileal total bladder ostomy. All the selected cases were summarized by retrospective analysis. According to the data obtained in this study, there are notable divergences in the occurrence of postoperative complications in different clinical stages after relevant statistical processing, with statistical significance. Finally, 40 patients were included, 27 males and 13 females, aged 57–81 (71.5 ± 5.5) years. They were divided into a continuous nursing group and a conventional nursing group, with 20 patients each with a simple randomization process. This method guarantees that each patient had an equal chance of being assigned to either group, thereby minimizing selection bias and ensuring that the baseline characteristics were comparable between the two groups. There was no notable divergence in age, sex, tumor stage, BMI, operation time, intraoperative bleeding volume, and hospital stay between the two groups (*P* > 0.05).

### 1.2 Method

#### 1.2.1 Routine nursing group

Routine nursing provides standard perioperative health education, a one-time stoma care training session at the bedside, and a single telephone follow-up one week after discharge without additional psychological counseling. Routine health education was carried out during the perioperative period, and stoma nursing was carried out at the bedside after the operation. Professional stoma nurses replaced 1–2 stoma bags, taught family members and patients about stoma nursing technology and complication prevention methods, and told them to replace one pocket by themselves. Stoma nursing manuals and discharge guidance notes were issued before discharge, and regular telephone follow-up was conducted one week after discharge.

#### 1.2.2 Continuous care group

Continuous nursing support over six months through repeated training sessions, home visits, and regular follow-ups every two weeks during the first three months and monthly thereafter. Furthermore, continuous nursing includes comprehensive psychological support and tailored patient education sessions. This study carried out continuous nursing work based on conventional nursing measures. The main contents were as follows: before the surgical treatment, the following three aspects should be done well, namely, preparation for the operation, the psychological education, and the observation of the condition. Observing the Patient's condition was mainly focused on mastering the Patient's case data and accompanying or guiding the Patient to complete various special and routine examinations before the operation. The implementation of psychological education was mainly based on active communication with patients. By combining the psychological conditions of patients and reasonably selecting communication methods, patients can grasp their conditions from the side or actively guide them to relieve their negative psychological state correctly and improve the degree of nursing cooperation. For surgical patients, the prevention and treatment of postoperative complications should be strengthened. At the same time, attention should be paid to the early rehabilitation exercise after the operation to maintain a healthy body posture. Based on these, we also need to give patients positive and effective emotional counseling which in turn helps strengthen the immune function of the body. In addition, it was necessary to make patients understand the importance of maintaining a good state of mind for disease recovery and regularly encouraged and comforted patients to help them build confidence. The pre-operative preparation was to ensure that the Patient's intestinal tract remained clean before total cystectomy and ileal bladder replacement. Instructed the Patient to start a liquid diet and avoid vegetables, fruits, and other foods with high fiber content on the day before the operation, and started drinking Polyethylene Glycol Electrolytes Powder + warm boiled water to empty the intestines at about 3:00 p.m. and closely observed whether the intestines of the Patient were clean or not. At the same time, the psychological state and sleep quality of the patients were understood the night before the operation. Patients with anxiety and difficulty sleeping could be given appropriate drugs to help them sleep. Measures such as clean enema, fasting, and water prohibition in the morning of operation day shall be taken to ensure that there were no feces and gas accumulation in the intestine before the operation to reduce the production of bacteria in the intestine and reduce the incidence of infection after the operation. During the operation, the operating room nurse closely observed the Patient's condition and actively cooperated with the operating staff. If necessary, venous catheterization or abdominal puncture catheterization should be performed to maintain circulation stability and strengthen anesthesia management to ensure smooth breathing, prevent hypoxia and carbon dioxide retention, and prevent pulmonary complications. After the operation, closely observed the Patient's living indicators and combined functional exercise with physiological, psychological, and dietary nursing. In addition, the drainage pipes for patients after surgery were usually left and right stent tubes, left and right pelvic drainage tubes, and urinary tubes. The nursing staff should fully grasp the placement position of each catheter and fix its position to avoid falling off, deformation, and folding, as well as to make the pipes unobstructed.

**Figure 1:**
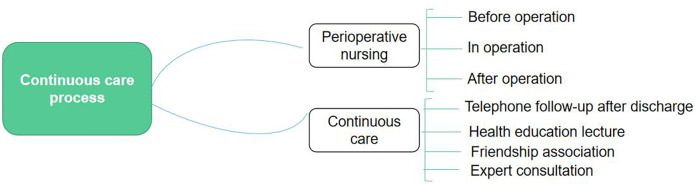
Continuous nursing process. This figure illustrates the continuous nursing process, detailing preoperative preparation, psychological support, postoperative care, and follow-up interventions. The figure highlights the continuous care interventions applied to patients.

Regular telephone follow-ups (once every two weeks before March and once every month from March to June) to understand the Patient's relevant information, answer the Patient's doubts, and provide relevant nursing guidance to encourage the patient to maintain good living habits and eating habits; Stoma nurses visited the patients' families in January, March, and June to help them solve stoma nursing and psychological problems; “Network follow-up” and “communication” (create a continuous stoma care WeChat group). Pushed stoma nursing knowledge every week, encouraged stoma nurses to share stoma nursing experience, and promoted interaction between stoma nurses. The stoma fraternity (once every March) was attended by professional stoma nursing personnel, stoma patients, and their families. It carried out stoma knowledge lectures, introduced new products and technologies, exchanged family nursing experience, encouraged patients to communicate with each other, and consulted on the spot (Figure 1).

### 1.3 Evaluation indicators

#### 1.3.1 Quality of life (QoL)

Half a year after the operation, the patients were followed up and collected information by telephone, WeChat, and out-patient follow-up. A bladder cancer-specific scale was used to evaluate patients' QoL. The scale compiled by Cella (20) of the Center for Outcome Research and Education (FACT-G) of Northwestern University in the United States included a standard module for measuring the QoL of cancer patients and a bladder cancer-specific module (BSS). FACT-G has 27 entries, including 4 parts of physical status, social/family status, emotional status, and functional status, and BSS contained 12 bladder cancer-specific entries. The higher the score, the higher the health-related QoL of the measurer. As a QoL scale, the validity of FACT-BL has been verified.

#### 1.3.2 Complications

The incidence of ileal bladder skin ostomy and skin-related complications were compared between the two groups half a year after the operation, and outpatient follow-up and photographic records were made. The main complications were peristomal inflammation, hemorrhage, edema, obstruction, and parahernia.

#### 1.3.3 Patient satisfaction

Within half a year after the operation, the third party sends the patients' satisfaction questionnaires (including very satisfied, satisfied, general, and dissatisfied questionnaires). The third party collected the survey results from the researchers and anonymously submitted them to the researchers to maximize the implementation.

#### 1.3.4 Gastrointestinal function

The gastrointestinal function scores of the two groups before and after nursing were compared.

### 1.4 Statistical analysis

SPSS 22.0 statistical software was used for data analysis. Accurate Fisher's test was used for secondary classification count, the likelihood ratio test for multiple classification count, measurement data were represented by (x ± s), and a *t*-test was used to compare groups. *P* < 0.05, with statistical significance.

## 2 Results

### 2.1 Qol score of two groups of patients

**Figure 2:**
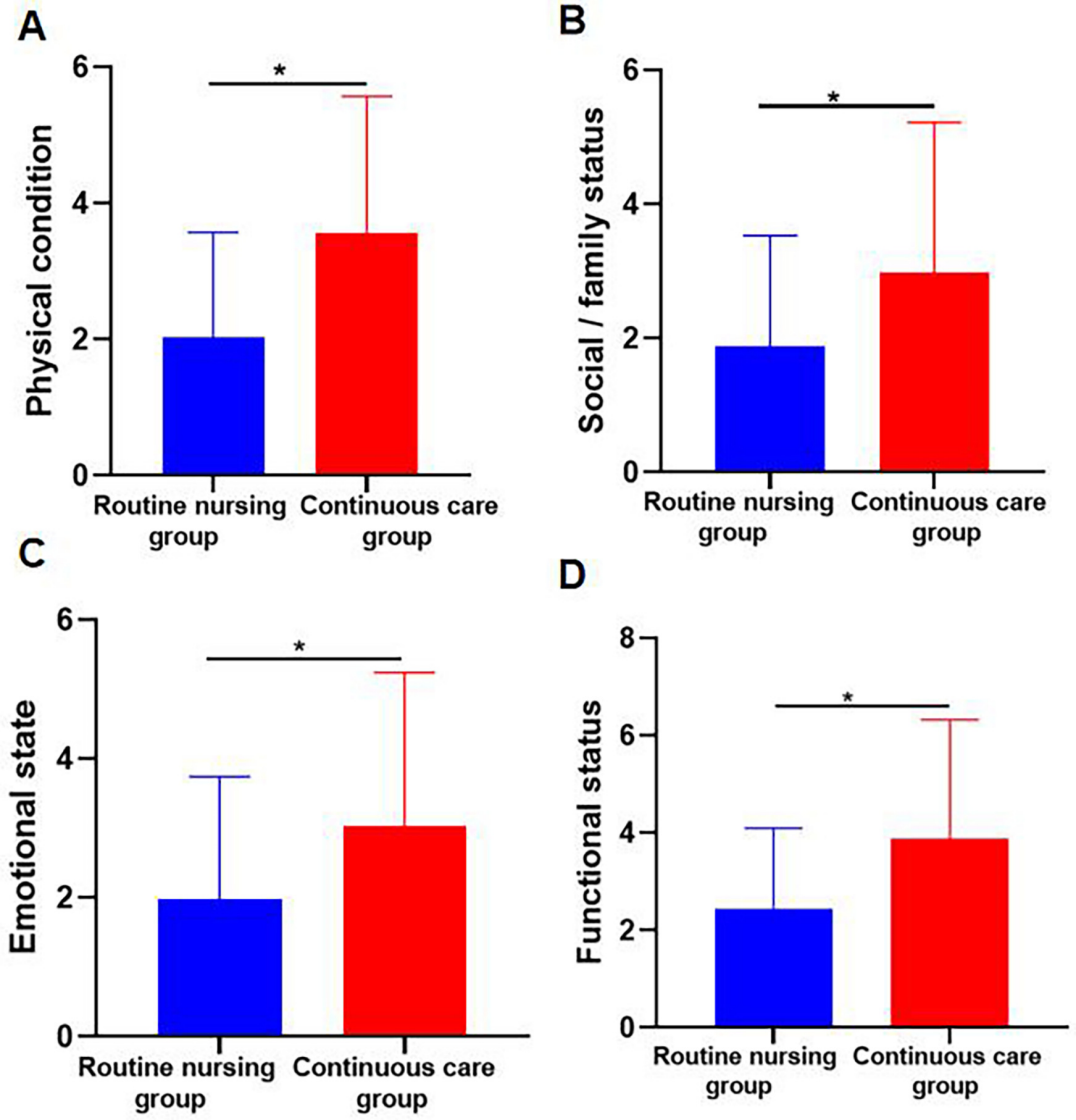
**(A)** Physical Status, **(B)** social/family Status, **(C)** emotional Status, and **(D)** functional Status. Data are presented as mean ± SD. An asterisk (*) indicates a statistically significant difference *(P* *<* *0.05)*. This figure presents the scores for physical status, social/family status, emotional status, and functional status for both the routine nursing group and the continuous care group. Data is shown for each domain, with statistical significance indicated by asterisks (*P < 0.05).

**Figure 3:**
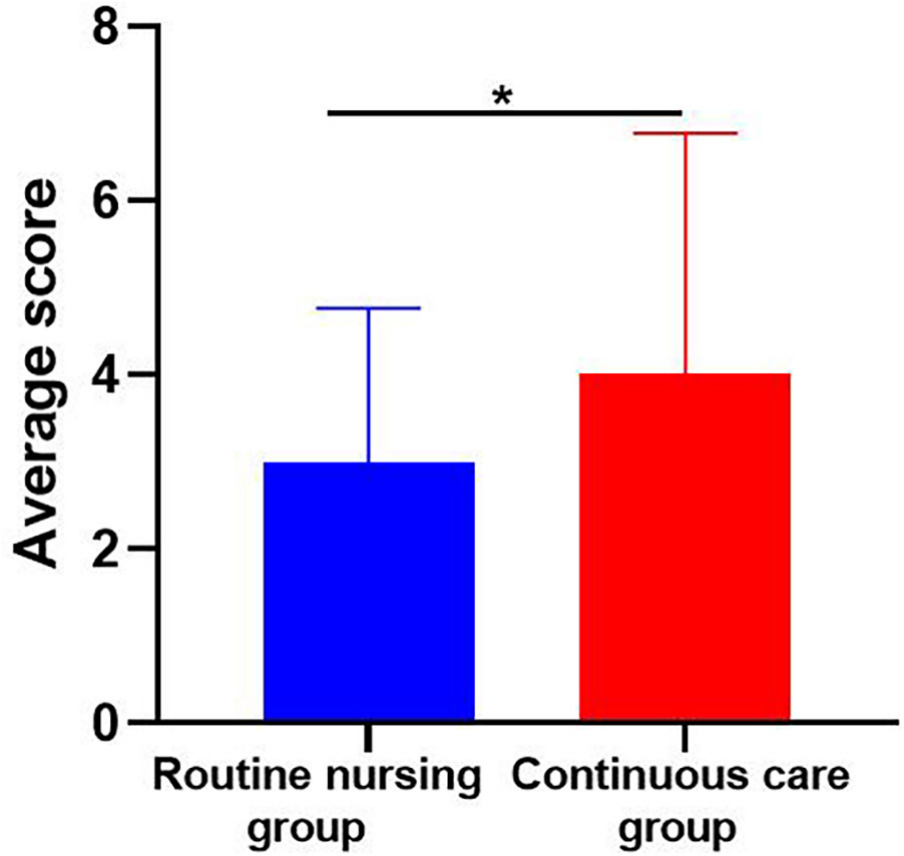
Overall QoL score comparison in the routine nursing and continuous care groups. *P* < 0.05 indicates statistical significance. This figure shows the average quality of life scores for the routine nursing group and the continuous care group. Differences between the groups are highlighted, with statistical significance indicated by an asterisk (*P *<* *0.05*).

The continuous nursing group scored higher than the conventional nursing group in four aspects: Physical status, social/family status, emotional status, and functional status. Shown in Table 1 and Figures 2, 3. Therefore, the QoL of the continuous nursing group was higher than that of the conventional nursing group.

**Table 1:** Qol scores in the routine and continuous nursing groups.

Group	Physical condition	Social/family status	Emotional state	Functional status	Average score
Routine nursing group	2.03 ± 1.54	1.88 ± 1.65	1.98 ± 1.76	2.45 ± 1.65	2.99 ± 1.78
Continuous care group	3.56 ± 2.01	2.98 ± 2.24	3.03 ± 2.21	3.88 ± 2.44	4.02 ± 2.76
*t*	4.236	3.165	4.998	4.786	5.034
*P*	*P* < 0.05	*P* < 0.05	*P* < 0.05	*P* < 0.05	*P* < 0.05

Higher scores indicate better QoL. Data are presented as mean ± standard deviation (SD).

*P* < 0.05 indicates statistical significance.

This table presents the Quality of life scores for patients in the routine nursing group and the continuous care group, assessed across four domains: physical condition, social/family status, emotional state, and functional status. Scores are expressed as means ± standard deviations. Statistical significance is indicated with *p*-values.

### 2.2 Comparison of complications and nursing satisfaction between the two groups

**Figure 4:**
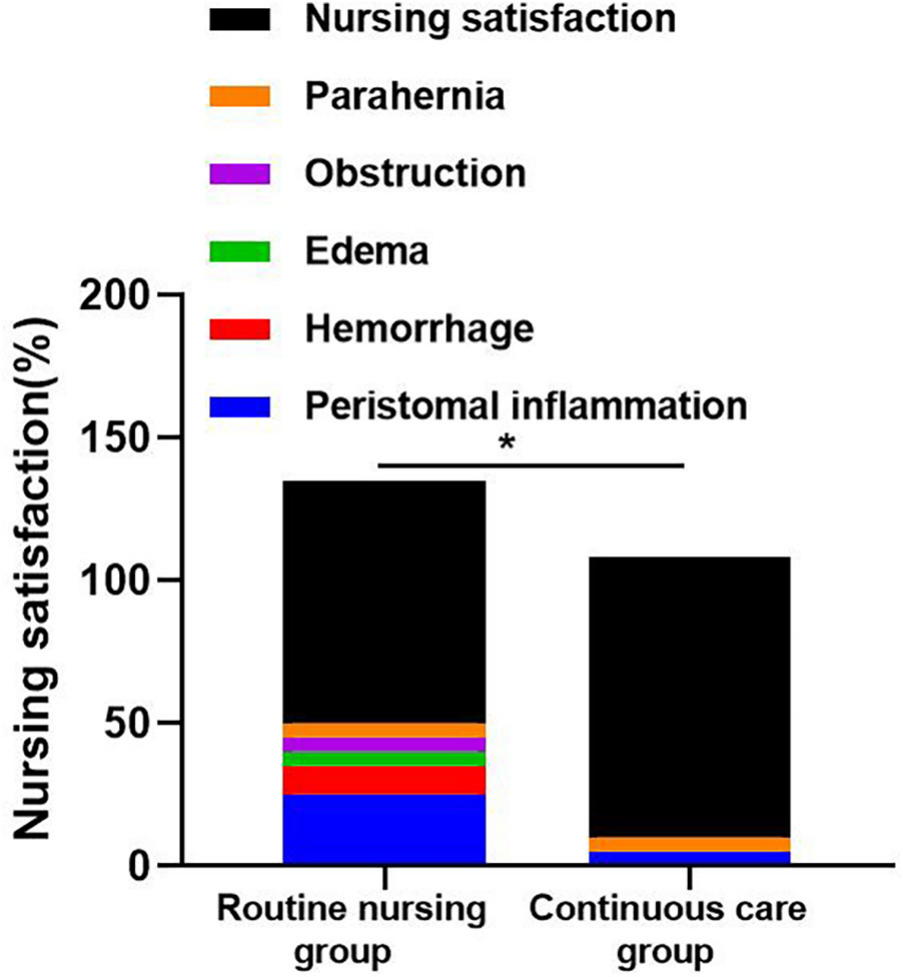
Comparison of post-surgical complications and patient satisfaction levels between the routine nursing group and the continuous care group. The continuous care group exhibited a lower complication rate and higher satisfaction. Data include peristomal inflammation, hemorrhage, edema, obstruction, and parahernia occurrence rates. Error bars indicate standard deviation. *P* < 0.05 indicates statistical significance.

There were 10 cases of complications in the routine nursing group, including 5 cases of peristomal inflammation, 2 cases of bleeding, 1 case of edema, 1 case of obstruction, and 1 case of parahernia; There were 2 cases of complications in the continuous nursing group, including 1 case of peristoma inflammation and 1 case of parahernia. Shown in Table 2 and Figure 4. The complication rate of the continuous nursing group was lower than that of the routine nursing group. The nursing satisfaction of the continuous nursing group was 98% higher than that of a routine nursing group (85%).

**Table 2:** Comparison of complications and patient satisfaction rates between groups.

Group	Peristomal inflammation	Hemorrhage	Edema	Obstruction	Parahernia	Nursing satisfaction
Routine nursing group	5 (25%)	2 (10%)	1 (5%)	1 (5%)	1 (5%)	85%
Continuous care group	1 (5%)	0 (0%)	0 (0%)	0 (0%)	1 (5%)	98%
*P*	*P* < 0.05	*P* < 0.05	*P* < 0.05	*P* < 0.05	*P* < 0.05	*P* < 0.05

Lower complication rates and higher satisfaction were observed in the continuous nursing group.

*P* < 0.05 indicates statistical significance.

This table compares the incidence of complications and nursing satisfaction between the routine nursing group and the continuous care group. It includes the frequency of peristomal inflammation, hemorrhage, edema, obstruction, and paraphernalia, as well as overall nursing satisfaction percentages. Statistical significance is indicated with *p*-values.

### 2.3 Gastrointestinal function scores of patients in both groups before and after nursing

**Figure 5:**
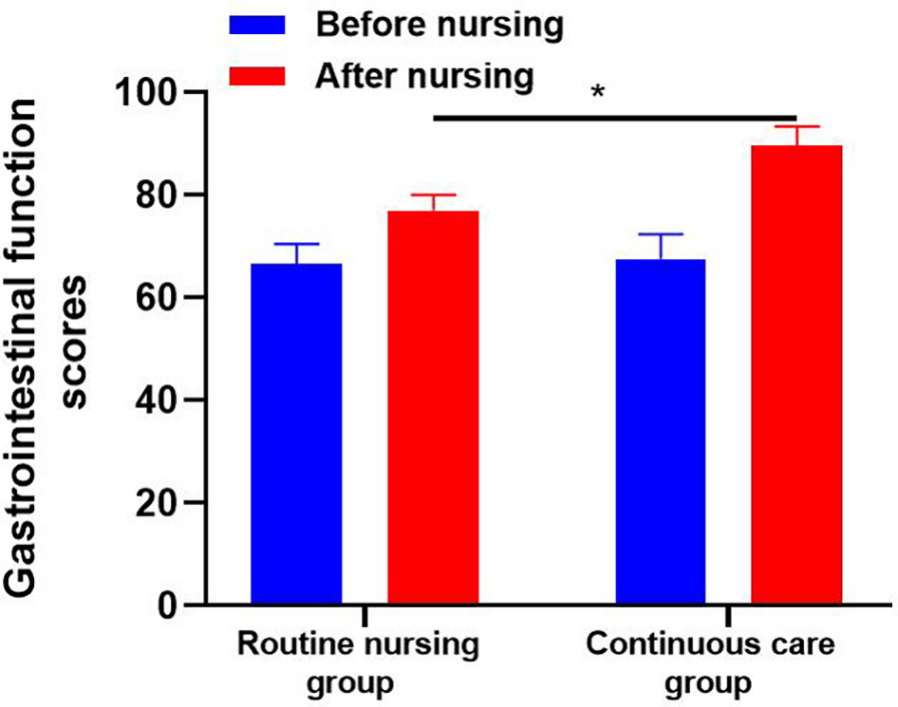
Gastrointestinal function scores before and after nursing interventions in the routine nursing and continuous care groups. The continuous care group demonstrated a greater improvement in gastrointestinal function compared to the routine nursing group. The statistical significance of the differences is indicated by an asterisk (*P *<* *0.05)*.

Table 3 and Figure 5 have complied gastrointestinal function scores of patients in both groups. The gastrointestinal function score of the routine nursing group was (76.96 ± 3.04), which was lower than that of the continuous nursing group (89.67 ± 3.67).

**Table 3:** Gastrointestinal function scores before and after nursing intervention.

Group	Before nursing	After nursing
Routine nursing group	66.65 ± 3.77	76.96 ± 3.04
Continuous care group	67.55 ± 4.86	89.67 ± 3.67
*P*	*P* > 0.05	*P* < 0.05

The continuous nursing group showed significant improvement.

*P* < 0.05 indicates statistical significance.

This table displays gastrointestinal function scores for patients in both the routine nursing group and the continuous care group, measured before and after nursing interventions. Scores are reported as means ± standard deviations. Statistical significance is indicated with *p*-values.

## 3 Discussion

Bladder cancer is a common urinary system tumor in the clinic. The incidence of bladder cancer in the world ranks eleventh among malignant tumors, and in China, it ranks first among urogenital system tumors. It is a disease that directly endangers the survival of patients (21–24). Radical cystectomy is still the gold standard for myometrial invasive bladder cancer. Urinary diversion or reconstruction is required after cystectomy, which will have a relatively significant adverse effect on the physiology and psychology of patients with stoma. In particular, patients with ileal bladder surgery face great pressure from psychological, physiological, and psychological aspects due to changes in their body image (25–27). In recent years, with the development of medical technology, more researchers have begun to pay attention to patients with bladder and urinary tract dysfunction (hereinafter referred to as “refractory urinary fistula patients”). Such people are often accompanied by anxiety and depression to different degrees (28–31).

It is important to study how to carry out effective nursing interventions for patients with such troubles to help them obtain more systematic nursing services. Continuous care ensures that patients can obtain different medical and psychological care levels in the hospital and the family by taking a series of nursing measures (32). It generally extends from the hospital to the family, including continuous follow-up and guidance in the discharge process, referral, and return to the family or community (33, 34). It is a new concept and term in the development of modern nursing science. It originated in the United States and has been widely used in many countries in Europe and America. With the continuous development of the social economy and the transformation of the medical model, continuous nursing has gradually been paid attention to by domestic scholars (35, 36). Saultz et al. (37) summarized the meaning of continuous nursing, mainly including the continuity of patient information, medical care services, and doctor-patient and nurse-patient relationships. Carrying out continuous nursing can promote communication between nurses and patients and enhance patients' trust in medical staff. In addition, continuous nursing helps reduce the incidence of complications, enhances the Patient's self-care skills, relieves mental pressure and destructive emotions, and lets the Patient's life get on the right track as soon as possible.

Previous research performed in Western healthcare environments (38, 39), where continuous nursing models are firmly established. our study demonstrates that organized continuous nursing interventions can provide similar advantages even in resource-constrained settings. This indicates that the incorporation of routine follow-ups, familial support, and network-oriented educational initiatives effectively mitigates difficulties and enhances QoL. In this study, continuous nursing significantly outperformed routine nursing in improving QoL, reducing complications, and increasing patient satisfaction.

Continuous stoma nursing takes family and patients as the core, and conventional stoma nursing measures as the main line to carry out continuous nursing work, mainly including regular telephone follow-up, Family follow-up of stoma nurses, “Network follow-up and communication” and “stoma fellowship” (40), who found that integrating an internet-based ostomy care platform with discharge planning significantly improved patient outcomes in colostomy patientsThese various forms of continuous nursing services, so that patients can receive continuous stoma care, health care, and psychological counseling and promote patients’ recovery when they are discharged (41). This provides multi-level humanized comprehensive nursing services for patients (42).

Our research demonstrates that continuous nursing markedly boosts QoL, diminishes stoma-related problems, and increases patient satisfaction in comparison to standard nursing practices. These findings align with recent research (43). Evidenced that continuous care interventions enhanced self-care capabilities and psychological well-being in stoma patients, resulting in elevated QoL scores.

This study found that the physical status, social/family status, emotional status, functional status, a specific score of bladder cancer, and total score of FACT-BL after continuous nursing were markedly better than those of the routine nursing group, which fully showed that family continuous nursing could markedly improve the QoL, of patients (19). In addition, through the comparative analysis of women of different ages, it is concluded that with the growth of age, the degree of disease cognition gradually decreases, but the emotional disorder is aggravated due to the deterioration of physical function. Therefore, corresponding countermeasures should be taken according to the change in age. The results have shown that the QoL of the patients in the continuous nursing service group was markedly better than that in the control group (44). Colostomy patients are greatly influenced by physiology and psychology after the operation. The QoL has declined markedly, and improving the QoL of patients after operation has become an essential purpose of continuous nursing.

The complication rate of the continuous nursing group was lower than that of the routine nursing group (45). There was no notable divergence in statistics, and there was a certain relationship with the number of patients enrolled. At the same time, from the discharge follow-up, there were no serious complications in both groups. However, regarding the number of complications and the absolute value of the incidence rate, the quality of family continuous nursing is higher than that of the routine nursing group, and the statistical divergence needs to be further confirmed by increasing the number of patients enrolled in the group. The survey (46) results showed that the incidence of stoma complications in the experimental group decreased as compared with the control group after the implementation of continuous nursing. These results are in alignment with the study by (47), showed that the implementation of routine microbiological surveillance in a specialized urological setting markedly decreased the incidence of nosocomial infections. This evidence highlights the importance of proactive, structured follow-up care. Moreover, due to the anatomical connection created by ileal cystostomy, bacterial translocation between the gastrointestinal and urinary systems is a critical concern. Studies have demonstrated that bacterial colonization from the ileum may contribute to urinary tract infections and peristomal complications postoperatively (48). Our findings affirm that continuous nursing interventions, which include targeted education, regular assessments, and psychological support, can help mitigate such risks by addressing both hygiene practices and early symptom management. In our study, the continuous nursing model which incorporated scheduled assessments, patient education, and ongoing support similarly contributed to a notable reduction in stoma-related complications, reinforcing the value of comprehensive postoperative care in improving clinical outcomes. This was similar to the survey results; it indicates that after continuous nursing, patients have a better understanding of stoma knowledge and nursing technology (49). It prevents and reduces the occurrence of complications, and takes the occurrence rate of stoma complications as the evaluation index of curative effect because reducing the occurrence of stoma complications in stoma patients is also the pursuit of continuous nursing (40).

In addition, continuous family nursing is the continuation of health education in the hospital and the practical action to establish a harmonious nurse-patient relationship (50, 51). Through various forms of continuous nursing, not only can health knowledge be taught, but patients can also experience the humanistic care of medical personnel, reduce the distance between nurses and patients, and increase Patient satisfaction. The results showed that the family continuous nursing group was more satisfied than the conventional nursing group. Although there was no notable divergence in statistics, satisfaction was increased, which shows that continuous care can be more affirmed.

In countries other than China, continuous nursing was carried out earlier, and various forms of nursing existed (35). A sound interdisciplinary medical service team carried out continuous nursing for patients when they were discharged, constituting a seamless connection between the hospital and the community. For example, countries like the United States and Canada have taken telephone follow-ups before discharge as a routine work mode; the UK began implementing the “family bed” in 2006. More than 500,000 families can accept this new care mode (52). After the stoma patient is discharged from the hospital, the specialist nurse will give detailed discharge guidance to the patient, contact the community service nurse, explain the main nursing problems and nursing plans of the patient, and the community nurse can solve the health needs of the Patient on time.

However, due to the current shortage of domestic nursing resources, especially the severe shortage of stoma nursing personnel in community medical institutions, most patients can't fully grasp the relevant nursing knowledge and technology after discharge and lack self-care ability (53). The way and means of receiving rehabilitation information and nursing after discharge are limited, which brings many inconveniences to the rapid recovery of patients and seriously affects the QoL (40). Although many Research reports have found that continuous family nursing has achieved remarkable results, it can't meet the current situation in China (54, 55). Therefore, China should still expand nursing manpower and add stoma nursing personnel in community medical institutions to carry out continuous stoma nursing better, promote the development of continuous nursing, and ultimately benefit patients. Although our study presents strong evidence for the benefits of continuous nursing, one of the limitations of this study is the relatively small sample size of 40 participants, which may affect the statistical power and generalizability of the findings. While the results indicate significant improvements in QoL, reduced complications, and enhanced patient satisfaction with continuous nursing, the limited sample size necessitates caution when extrapolating these outcomes to broader populations. Future studies with larger, more diverse cohorts are needed to validate these findings and provide a more comprehensive understanding of the impact of continuous nursing on patients undergoing ileal cystostomy. Additionally, multi-center trials could enhance the robustness of the data and support the development of evidence-based guidelines for clinical practice.

## 4 Conclusions

This study highlights the significant impact of a continuous care model in improving patient outcomes during treatment. The findings demonstrate that patients who received structured, ongoing support exhibited higher satisfaction levels, reduced anxiety, and better adherence to treatment protocols compared to those receiving standard care. These results underscore the importance of integrating personalized and sustained care approaches into routine clinical practice. The novelty of this study lies in its systematic implementation of a continuous care framework tailored to patient needs, which not only enhanced clinical outcomes but also fostered a more responsive and empathetic healthcare experience.

## Data Availability

The original contributions presented in the study are included in the article/Supplementary Material, further inquiries can be directed to the corresponding author.
